# Non-communicable disease care in peri-urban Nepal: Potential for community-based interventions

**DOI:** 10.1371/journal.pgph.0005015

**Published:** 2026-07-15

**Authors:** Maryam Hameed Khan, Yoko Inagaki, Sonam Magar, Sweta Koirala, Nisha Rana, Pabitra Babu Soti, Gabriella Rose Chalker, Kamal Ghimire, Svea Closser, Dinesh Neupane

**Affiliations:** 1 Department of International Health, Johns Hopkins Bloomberg School of Public Health, Baltimore, Maryland, United States of America; 2 Nepal Development Society, Kathmandu, Nepal; 3 Middlebury College, Middlebury, Vermont, United States of America; University of Sydney, AUSTRALIA

## Abstract

Non-communicable diseases (NCDs) are a growing public health challenge in Nepal, driven by hypertension, diabetes, and smoking, and contributing substantially to morbidity and mortality. Access to equitable and affordable care remains limited, particularly in community settings. Task-sharing with Female Community Health Volunteers (FCHVs), a national cadre of volunteer community health workers primarily engaged in maternal and child health, offers a potential strategy to improve community-based NCD management, but its feasibility requires careful assessment. During the formative phase of the SCALE-NCD project, this qualitative study explored community and health system perspectives on NCD care and community-based delivery models in Pokhara, Nepal. Data were collected through 17 in-depth interviews and six focus group discussions with community members, FCHVs, facility-based community health workers (FB-CHWs), and representatives from government, public health, academia, and telecommunications. Thematic analysis revealed five core findings. Community members recognized NCD risk factors but reported deep mistrust in government health services driven by negative care experiences, financial, and structural barriers. Perceptions of FCHVs were shaped by limited community exposure to their roles beyond maternal and child health. While some participants questioned FCHVs’ capacity to manage NCDs, others valued their familiarity and accessibility when services were reliably supported. FCHVs and FB-CHWs emphasized that infrequent training, limited supervision, chronic stockouts, financial strain, and inconsistent incentives undermined service delivery and FCHV motivation. Stakeholders stressed that sustainable integration of community-based NCD care would require government resourcing, local ownership, and alignment with existing systems. Participants expressed cautious interest in mHealth strategies, including SMS and audio messages, to support awareness and follow-up. These findings informed the final design of SCALE-NCD, a multi-component task-sharing intervention, and underscore the importance of strengthening FCHV training and supervision, ensuring supplies and incentives, and building community trust through consistent, locally supported service delivery when scaling community-based NCD programs in similar resource-limited settings.

## Introduction

Non-communicable diseases (NCDs), including hypertension, diabetes, and smoking-related illnesses, are a growing public health burden in low- and middle-income countries (LMICs), where health systems often struggle to provide effective long-term management [1]. In Nepal, NCDs account for 71% of total deaths, with hypertension, high fasting blood glucose, and smoking among the leading risk factors for morbidity and mortality [2]. Despite the rising burden, Nepal’s health system remains primarily oriented towards maternal and child health.

A key challenge in NCD care is the health workforce gap, particularly in LMICs where physician-to-patient ratios are low [3–5]. Task-sharing approaches, where community-based providers take on expanded roles in prevention, screening, and referral, offer a promising strategy to bridge these gaps in NCD care [6–9]. Globally, sharing tasks with community health workers (CHWs) has played a pivotal role in extending essential services for infectious diseases and maternal and child health in LMICs [10]. As the burden of NCDs has grown, this approach has increasingly been expanded to chronic disease prevention and management. Across diverse LMIC settings, CHWs have supported community-based NCD care through screening for hypertension and diabetes, assessment of tobacco use, and lifestyle counseling [6,7,11].

Within this global context, Nepal’s Female Community Health Volunteers (FCHVs)— a nationally deployed cadre of community health workers— represent a workforce embedded within their communities and experienced in health promotion. FCHVs are local women who serve on a voluntary basis and are not salaried by the government, although they may receive small allowances for specific activities [3]. Previous research has demonstrated that FCHVs can effectively contribute to NCD care by measuring blood pressure, providing counseling, and referring high-risk individuals [3,12–14]. However, despite growing interest in integrating FCHVs into NCD management, evidence on their role in long-term NCD care remains limited, particularly regarding expanding responsibilities, and the social and structural barriers to sustainable implementation [3,15,16]. Equally important are the broader structural and contextual factors that shape the success of community-based NCD interventions. For example, trust in government health systems has been identified as a key determinant of uptake and adherence to care in Nepal. Studies suggest that communities with low confidence in public health facilities are less likely to utilize services, adhere to treatment, or follow referrals, which may limit the impact of community health interventions. Understanding these dynamics is therefore essential when integrating FCHVs into chronic disease management programs.

In addition to task-sharing, mobile health (mHealth) interventions, such as SMS reminders and phone call follow-ups, have demonstrated potential in supporting chronic disease management [17]. However, their implementation in LMICs requires careful consideration of contextual factors. In Nepal, where digital literacy and phone ownership vary widely, early studies suggest potential for mHealth strategies, but evidence on their broader feasibility and acceptability in NCD management remains limited [18,19]. To design effective and scalable NCD interventions, it is critical to assess whether mHealth approaches can complement community-based health services.

This study presents findings from the formative, pre-implementation phase of the ‘Scaling Up Community-based Noncommunicable Disease Research into Practice in Pokhara Metropolitan City of Nepal’ (SCALE-NCD) project, a hybrid type 2 effectiveness-implementation study evaluating a task-sharing and mHealth intervention. FCHVs deliver community-based care for hypertension, type 2 diabetes, and tobacco smoking, complemented by SMS reminders to reinforce counseling and support follow-up. Using qualitative data from diverse community and health system stakeholders, this paper explores the structural, social, and contextual factors that may influence the design, delivery, and sustainability of community-based NCD interventions involving FCHVs. Specifically, it examines: (1) barriers and facilitators to FCHV-led NCD management, including health system trust, training, and compensation; and (2) the feasibility and acceptability of complementary mHealth strategies to support community-based care. By identifying these factors, the study seeks to inform the development of scalable, contextually appropriate task-sharing models for chronic disease prevention and management in peri-urban metropolitan settings in Nepal and similar LMIC settings.

## Methods

### Ethics statement

The study received ethical approval from the Institutional Review Boards of the Johns Hopkins Bloomberg School of Public Health and the Nepal Health Research Council. Written informed consent was obtained from all participants prior to data collection. Additional information regarding the ethical, cultural, and scientific considerations specific to inclusivity in global research is included in the Supporting Information (S1 Questionnaire).

This qualitative study was conducted in Pokhara Metropolitan City, Nepal, in partnership with the Nepal Development Society (NeDS), a local community-based research and implementation organization. The study area comprises both peri-urban and rural communities with mixed reliance on public and private health systems.

Participant recruitment and data collection took place from 11/11/2024–24/01/2025 through focus group discussions (FGDs) and in-depth interviews (IDIs) with key stakeholders. Inclusion criteria for community members included residing in the study area and having at least one NCD risk factor (hypertension, diabetes, or current tobacco use). FCHVs and facility-based CHWs were eligible if they were actively providing health services in the study area. Key organizational representatives were selected based on their role in NCD programming, health policy, or digital health initiatives. A purposive and convenience sampling strategy was used to recruit participants who could provide diverse insights into the feasibility and acceptability of FCHV-led NCD management and mHealth interventions, either as users, providers, or system actors. Sampling was designed to reflect a range of socioeconomic, geographic, and health system perspectives. Recruitment was led by trained research staff from the Nepal Development Society (NeDS) with assistance from local health officials and community leaders. Participants were contacted by phone and in person, informed of the study objectives, and invited to join an FGD or IDI at a convenient location. Four target groups were included:

1. Community members with NCD risk factors (i.e., hypertension, diabetes, or smoking) (five FGDs)
2. FCHVs (two IDIs, one FGD for a total of nine participants)
3. Facility-based community health workers (FB-CHWs) (six IDIs)
4. Representatives from key organizations, including government health agencies, local public health offices, academic institutions, international organizations, and telecommunications and advocacy sectors (nine IDIs)

Data collection continued until thematic saturation was reached within and across participant groups, defined as the point at which no new themes or sub-themes were emerging from interviews or focus groups.

Semi-structured interview guides tailored to each participant group were used to collect data. The guides covered the following key topics:

- Community perspective of NCD risk factors and health seeking behavior
- Trust in the government health system
- Perceptions of FCHVs’ role in NCD management
- Health system constraints to community-based NCD care
- Feasibility of mHealth strategies

A total of 6 FGDs and 17 IDIs were conducted by trained qualitative researchers in Nepali. FGDs allowed for group-level discussions and collective insights, while IDIs provided an opportunity for in-depth exploration of individual experiences and perspectives. Interviews were held at the NeDS office, health posts, or respondents’ homes in Nepali and lasted between 30 minutes and two hours. Written informed consent was obtained from all participants. Audio recordings were transcribed verbatim, translated into English, and supplemented by field notes, which were integrated into the analysis to enhance interpretation and contextual understanding.

Thematic analysis was conducted using NVivo software. The analysis followed both an inductive and deductive approach. An initial codebook was developed based on study objectives and relevant literature, and themes were iteratively refined to reflect emerging patterns in the data. Deductive coding was informed by the study’s objectives to identify themes related to feasibility and implementation outcomes, and inductive coding allowed for the identification of emerging themes based on participant narratives. Two independent researchers coded the data to ensure inter-coder reliability, and discrepancies were resolved through discussion. Findings were discussed in relation to key cross-cutting factors identified in the WHO guidance on optimizing health worker roles through task shifting for maternal and newborn health interventions [20]. These factors — governance and leadership, access to commodities, service delivery, health workforce training, and financing — are broadly applicable to community-based task-sharing interventions and provide a useful lens for interpreting our qualitative findings on FCHV involvement in NCD care.

## Results

### 1. Community composition

The study sites were located approximately 30–45 minutes from downtown Pokhara and encompassed communities with varying degrees of urbanization, ranging from densely populated settlements to more sparsely populated peri-urban and rural areas. Participants described a socially and economically diverse population, including households engaged in farming, small businesses, government employment, and foreign labor migration.

Respondents highlighted socioeconomic diversity within and across communities, noting that economic status was shaped not only by caste or ethnicity but also by land ownership, migration histories, and employment opportunities. While some households had accumulated wealth through foreign employment or business activities, others faced ongoing economic hardship.

Migration emerged as an important contextual factor shaping community composition and service delivery. Many younger individuals had migrated abroad for work, leaving behind older residents who often managed household or agricultural activities. In some areas, participants described entire households relocating, resulting in communities characterized by both ongoing urbanization and population decline.

These demographic and socioeconomic differences provided important context for understanding variations in NCD risk, healthcare access, and the potential implementation of community-based interventions described in subsequent sections.

### 2. Community perspectives on NCDs

#### Perception of NCD risk factors.

Participants generally demonstrated awareness of major NCD risk factors. Across stakeholder groups, NCDs were primarily understood as consequences of lifestyle behaviors, with many participants linking disease onset to changing diets, sedentary routines, and reduced physical labor associated with urbanization. Processed and pesticide-laced foods were seen as unavoidable due to reliance on market produce, and excessive consumption of salt, oil, and meat was commonly cited as a risk factor. Participants frequently described these exposures as difficult to avoid within their current living environments. A few participants felt lower-income individuals were at risk due to limited awareness of preventive health measures and difficulty accessing health services.

Beyond behavioral risk factors, many participants also highlighted broader social and contextual drivers of NCDs. Stress associated with family responsibilities, financial pressures and employment were commonly viewed as contributing to hypertension. Some linked NCD risk to labor migration experiences, particularly physically demanding work abroad, while others emphasized hereditary susceptibility.

Overall, participants emphasized that NCDs were increasingly affecting all segments of the community.

#### Barriers to management.

Despite relatively high awareness of NCDs, we saw that awareness did not necessarily translate into care-seeking or preventive action. Participants identified several barriers that limited screening uptake, timely treatment initiation, and long-term disease management.

***I. Concerns about treatment*:** Misconceptions about chronic disease treatment emerged as an important barrier to care. Health workers described a widespread belief in the community that initiating hypertension medication created lifelong dependence, leading some individuals to delay treatment despite elevated risk.

Concerns about side effects and perceived harms further contributed to reluctance to begin or maintain treatment. A community member commented in an FGD, *“When you take medication for diabetes, it leads to other health problems... Diabetes medication causes other diseases. It’s very harmful.”*

***II. Hesitancy to screening*:** Participants reported that screening was not universally accepted, particularly among individuals who did not perceive themselves to be ill. FCHVs described resistance from community members who doubted the reliability of screenings, and questioned the value of screening in the absence of immediate treatment.

Stakeholders emphasized the high proportion of undiagnosed NCDs. A public health officer described findings from screening camps in Pokhara where 3–5% of attendees were newly diagnosed with hypertension or diabetes, and another confirmed the high burden of cases in communities. He noted *“Whatever community we go to … a lot of hypertension patients can be found who are not taking medicine”,* and another added that large portions of the population *“remain hidden from the health systems and untreated.”*

***III. Delayed healthcare-seeking behavior*:** Even when diagnosed with hypertension or diabetes, some individuals remained skeptical of their test results, particularly due to the often-asymptomatic nature of chronic diseases. Health workers noted that some patients outright refused treatment because they did not feel unwell. Community members mentioned that they only sought checkups when they experienced symptoms such as dizziness or discomfort, rather than proactively monitoring their condition at regular intervals. Some admitted they did not monitor at all, relying instead on medication to keep their condition stable. An FCHV shared, *“Occasionally, when a patient’s BP is extremely high, like 200 or 210, and we provide treatment, they might say, ‘I don’t feel any symptoms. You’re saying my BP is this high, but I don’t believe it.’”*

Concerns about test accuracy and differing measurements across facilities further contributed to uncertainty regarding diagnosis and treatment decisions. Some community members felt that readings taken at local medical stores were unreliable, leading them to question their diagnosis or delay follow-ups. One said in an FGD, *“They might say, ‘It’s 140/90,’ but when I go to a proper doctor or hospital later, it turns out to be 160/95.”* Several FCHVs described cases in which community members ignored referrals despite markedly elevated blood pressure or glucose readings, highlighting challenges in translating screening into timely care-seeking.

***IV. Financial barriers and access to care*:** Financial barriers emerged as a cross-cutting challenge affecting both diagnosis and long-term disease management. Participants described difficulties affording consultations, follow-up visits, and medications, particularly when treatment required ongoing expenditures. These barriers were compounded by medication shortages within government facilities, which often forced patients to seek medicines through private pharmacies at higher cost.

#### Treatment adherence.

Upon diagnosis, some individuals reported reducing salt and sugar intake, exercising regularly, and avoiding alcohol. However, long-term adherence to lifestyle modifications was frequently described as challenging, with participants reporting gradual return to previous habits over time, despite awareness of their health conditions.

In contrast to lifestyle modifications, medication adherence appeared relatively strong among many diagnosed individuals. Many participants reported taking their prescribed medication consistently for several years, suggesting acceptance of pharmacological treatment once initiated. However, some individuals mentioned missing doses or discontinuing treatment because of cost, medication shortages, perceived improvement in symptoms, or personal oversight.

These findings suggest that awareness of NCD risk factors alone may be insufficient to promote effective disease management. The challenges in managing NCDs were further compounded by widespread perceptions and experiences that shaped community trust—or mistrust—in the health system. While our findings highlight evolving risk awareness within the community, they also raise a critical question: where do individuals turn when they seek care?

### 3. Health system trust

#### Government health services.

Trust in government health services emerged as a major factor shaping healthcare-seeking behavior and perceptions of community-based NCD interventions. Across participant groups, distrust was driven by concerns about availability of medications, perceived quality of care, financial barriers, and negative experiences within public facilities. While a few participants noted improvements in certain facilities, there was generally a deep-rooted distrust in the public healthcare system.

***I. Medication and supply shortages*:** Participants consistently identified medication and supply shortages as one of the strongest contributors to mistrust in government health services. Frequent stockouts, limited medication quantities, and the need to purchase medicines privately undermined confidence in the ability of government health facilities to provide reliable long-term care for chronic conditions.

Healthcare workers and health system stakeholders similarly described medication shortages in those facilities as reflecting broader limitations in system readiness and service delivery capacity. One government health worker explained, *“At that time, there was a severe shortage of medicines. Even if we diagnosed the issue [health condition], we didn’t have the capacity for first-line treatment at our level.”*

Concerns also extended to the perceived quality of free government-issued medications, with some community members believing they were not as effective as those purchased privately. A community member commented in an FGD, *“The health post doesn’t provide very good medicine, they give cheap medicine.”*

A few participants reported instances where doctors at government hospitals discouraged the use of government-provided medications. Some also mentioned that government doctors informally directed them to seek care at those doctors’ private practices instead.

While distrust was the dominant sentiment, a few participants noted some improvements in government health services. One individual shared, *“I also saw some good antibiotics there [health post] recently. When I asked, they said they just started providing it. If they consistently provide good medicines, people might start believing in the health post again.”* Others concurred that they might visit health posts more often if their preferred medicines were always available.

***II. Financial barriers and insurance challenges*:** Participants described financial barriers persisting even within publicly funded services. Although Nepal’s government health insurance scheme provides subsidized medicines and improved access for some individuals, many participants reported difficulties obtaining needed medications, uncertainty about covered services, and concerns regarding transparency in medicine distribution. A participant noted, *“They provide cheaper medicines but not the expensive ones. For example, they don’t give medicines that cost 24–25 rupees per tablet.”*

For uninsured individuals, the financial burden was even greater, leading some to borrow money or take loans to afford basic prescriptions. These experiences contributed to perceptions that government services were unreliable, particularly for individuals requiring ongoing NCD management.

***III. Patient experience*:** Interactions with healthcare providers strongly influenced perceptions of trust. Many participants described government facilities as difficult to navigate, citing long wait times, poor communication, and perceived disrespectful treatment. As one participant recounted, *“Today, for example, you have to stand in line to get the ticket, and the timing is never convenient. Government places are even less punctual. The doctor doesn’t come until 10:30 AM. Then, after getting the ticket and running around from one counter to another, you won’t finish before 3 PM”.*

Some also reported experiences of unequal treatment at government facilities based on socioeconomic status. *“They don’t do anything there; they only discriminate between the rich and the poor,”* a participant from a rural community stated. Some accounts were particularly severe, with community members particularly from rural populations in the peri-urban areas expressing strong distrust in certain facilities. *“The health post is there… you saw it. They try to kill people there,”* a respondent commented.

Collectively, these experiences reflected perceptions that public facilities were less responsive and less patient-centered than private providers, discouraging utilization of government services.

#### Private health facility preference.

Due to persistent frustrations with government health services, many participants expressed a strong preference for private providers despite the financial burden. Private facilities were perceived as offering shorter wait times, better communication, greater continuity of care, and more convenient access to services. Community members often described longstanding relationships with private providers and viewed these relationships as an important source of trust and continuity in chronic disease management.

However, participants also acknowledged that private care was not financially accessible for everyone. Lower-income households often remained dependent on government facilities despite concerns regarding service quality, highlighting the constrained choices faced by many community members.

#### Workload and motivation: FB-CHWs.

While community members expressed concerns about quality of care and tended to blame facility staff for those shortcomings, facility-based CHWs (FB-CHWs) emphasized the broader system constraints that limited service delivery. They described health posts as operating with limited staffing, while simultaneously managing clinical care, outreach activities, infectious disease control programs, reporting responsibilities, and administrative tasks. An FB-CHW described the strain of working with a limited team, *“We are two [at the health post]… We do feel work overload. There is insufficient staff… One has to manage OPD services, another has to manage family planning and antenatal checkups. We also have to manage the administrative part. So, there is a massive workload.”*

Rising demand for hypertension and diabetes services was perceived as adding to this already substantial workload. Although some government health post staff viewed workloads as manageable, many FB-CHWs emphasized that participating in new NCD interventions would be difficult without greater workforce support. Participants at government health posts, citing these staffing shortages, suggested that community-based approaches through FCHVs might be more feasible mechanisms for extending NCD services than interventions based in facilities.

Despite these challenges, FB-CHWs generally expressed strong commitment to serving their communities. However, they raised the need for adequate human resources, timely salary payments, and organizational support if they were to have sustained engagement in future community-based NCD interventions.

### 4. FCHVs in NCD management

#### Trust and credibility.

Community members generally recognized FCHVs as familiar figures in their neighborhoods due to their longstanding involvement in maternal and child health activities, vaccination campaigns, and health education initiatives*.* However, many participants noted that FCHVs primarily focused on child health, with limited involvement in addressing adult healthcare needs.

Under Nepal’s national health system, FCHVs are formally responsible for delivering maternal, newborn, and child health services. NCD-related tasks, such as screening and counseling for hypertension or diabetes, are not part of their standard responsibilities, although some FCHVs have been engaged in such activities through time-limited pilot programs led by NGOs or other partners. Consequently, community exposure to FCHVs’ role in NCD care was shaped by whether such external programs were active in their area; perceptions ranged from strong support to skepticism.

Across stakeholder groups, trust in FCHVs’ expanded role appeared closely linked to perceptions of competence, which in turn was linked to exposure to FCHVs carrying out certain tasks. While some community members questioned whether FCHVs possessed the skills necessary to conduct blood pressure and glucose screening, FCHVs described how community confidence in such activities increased over time when screening results were subsequently confirmed at health facilities. *“Many people would claim they didn’t have sugar [diabetes] or high blood pressure,”* one FCHV commented, adding, *“But when we tested them and referred them to health institutions, they found out they actually had those conditions. That’s when they started believing us… They came back and thanked us for detecting their issue.”* FCHVs also noted that their constant presence in the community made them well-positioned to provide early detection and referrals.

Participants also emphasized that for FCHVs to be accepted as care providers for NCDs, they needed to be able not only to conduct screenings, but also to explain results, provide counseling, and guide individuals toward appropriate care. *“They need to detect the disease, check the sugar, check the blood pressure… If medication is required, if the disease needs to be explained—everything. Just tying a device and taking it off is not enough,”* a community member shared. Several stakeholders noted that trust in FCHVs appeared stronger in rural communities than among wealthier or more educated urban households who might be less receptive to volunteer-led care.

#### Accessibility and community reach.

FCHVs, community members, and health system stakeholders consistently highlighted the unique position of FCHVs within their communities. Their continuous presence, familiarity with local households, and established relationships were viewed as important advantages for NCD prevention and management. *“People really look at FCHVs positively... they have worked in maternal health, so now people look at them with respect,”* a public health officer noted.

Community members suggested that regular home-based screening and counseling could improve early detection of hypertension and diabetes. Similarly, FB-CHWs emphasized that FCHVs played a critical role in community mobilization, noting that they often succeeded in reaching households that formal health services struggled to engage. Participants frequently described FCHVs as more accessible than facility-based providers. As one FB-CHW explained, *“FCHVs are available 24/7. But we only operate between 10 AM to 5 PM, so in terms of timing, they are always accessible, morning or evening,”*

Overall, FCHVs were perceived as an important bridge between communities and formal health services.

#### Prerequisites for expanding FCHVs’ role.

Although participants generally supported greater FCHV involvement in NCD care, many viewed such expansion as contingent on adequate training, equipment, and integration with the formal health system.

Many participants expressed concerns about FCHVs’ medical knowledge and ability to perform NCD-related tasks independently. FB-CHWs often viewed FCHVs as effective mobilizers and educators but questioned the extent of their clinical role, suggesting that community acceptance of FCHV-led NCD services would depend on their ability to conduct screening accurately and provide appropriate guidance and referrals.

At the same time, several stakeholders pointed to previous training experiences as evidence that FCHVs could successfully acquire NCD-related skills when provided with adequate support. Consequently, stakeholders repeatedly emphasized the need for sustained investments in capacity building. Community members, FB-CHWs, and FCHVs highlighted access to blood pressure monitors and glucometers, refresher training, supportive supervision, and stronger linkages with health facilities as key requirements for expanding FCHV-led NCD services. As one FB-CHW noted, *“They would benefit if they had their own tools to measure blood sugar and blood pressure. If they were trained and equipped to do so, they could measure these for people nearby.”*

#### Sustainability of volunteer model.

While support for FCHV involvement in NCD management was widespread, concerns about long-term sustainability emerged across stakeholder groups. Participants noted that NCD prevention and management would require ongoing screening, follow-up, and patient engagement, raising questions about whether a volunteer workforce could sustain expanding responsibilities over time.

Stakeholders highlighted several challenges, including the aging FCHV workforce, difficulties recruiting new volunteers, and concerns about educational preparedness for increasingly complex health responsibilities. One participant noted that *“Older volunteers have retired... and the center is not recruiting new ones.”* And another shared, *“Looking at sustainability, FCHVs might not last long because most of them are not highly educated. In the future, we might not get anyone ready to be FCHVs, as people may not want to volunteer anymore.”* Participants proposed potential policy solutions to address growing burden of NCDs, including the recruitment of replacement volunteers, deploying Auxiliary Nurse Midwives (a type of FB-CHW) at the community level and expanding the model to include male community health volunteers.

Overall, while there was broad support for involving FCHVs in NCD management, several structural and systemic challenges were seen as critical to address before scaling such approaches. The following section unpacks these implementation barriers, including workforce constraints, training, and compensation and sustainability.

### 5. Health system constraints to community-based NCD care

#### Compensation.

Participants consistently identified compensation as a major constraint to sustaining FCHV involvement in community-based NCD care. While FCHVs were committed to serving their communities, they emphasized that the current system often created financial strain and did not adequately reflect the time, effort, and opportunity costs associated with their community health work. Attending trainings, conducting home visits, and participating in health activities often required them to forego agricultural work or other income-generating opportunities.

Several FCHVs noted that resource limitations affected service delivery directly. For example, some FCHVs reported being unable to use personal funds for patient follow-up through phone calls. *“If we need to make calls, perhaps a provision for a SIM card or phone expenses could be made. If we are working voluntarily, it’s difficult to spend from our own pockets,”* one FCHV commented.

Stakeholders emphasized the need for stronger incentive structures to maintain FCHV motivation and engagement in expanded tasks. A hospital superintendent added*, “They are not satisfied. Their daily complaint is that they are not satisfied with what they got… The motivation to engage them is financial. That’s what’s needed now.”*

While many participants felt that transitioning FCHVs to salaried positions might not be feasible, financial and non-financial incentives including transport support, insurance coverage, free healthcare, and public recognition were proposed as mechanisms for sustaining motivation. One stakeholder commented, *“We can’t give them salary… but we must motivate them through some form of incentives. Free insurance, free medicine, or even lunch and transport costs during program days — these things should be offered.”*

#### Training and resource limitations.

Participants generally supported expanding the role of FCHVs in NCD management but emphasized that training alone would be insufficient without reliable access to equipment, medicines, and ongoing support. Both FCHVs and FB-CHWs reported receiving some NCD-related training through programs in the past, yet many described these past initiatives as infrequent, limited in scope, or discontinued after the initial rollout.

Stakeholders repeatedly highlighted the importance of refresher training, supportive supervision and stronger integration with health facilities. Participants emphasized that newly acquired skills could not be effectively translated into practice when essential supplies were unavailable. FB-CHWs described frequent and prolonged shortages of glucometers, test strips, medications, and other commodities, while FCHVs reported lacking functioning equipment needed to conduct community-based screening and follow-up. *“When it [medication] is out of stock, do patients still come in? Yes. Do they scold us? Yes. They say, ‘You didn’t ask for it [medication supply].’ But we did.”*

Training effectiveness was linked to broader health system readiness. *“Sometimes, we even question whether we should continue with this work. How can we come closer to making this system work? If you provide training, ensure availability of common medicines for sugar and blood pressure patients at these posts. If such systems were in place, things would improve a lot.” An FCHV commented.*

Workforce capacity and commodity availability were viewed by many respondents as interdependent requirements for successful implementation of community-based NCD interventions.

#### Sustainability and government ownership.

Participants also emphasized that long-term sustainability of task-sharing with FCHVs for NCD care would depend on integration within existing government systems rather than reliance on short-term external support. As one policymaker noted, *“The most important aspect of sustainability is integrating it [task-sharing intervention] into the system.”* He added, *“The main thing is to use the evidence to convince policymakers since they are the ones who ultimately implement it. Health involves various partners, and we need to demonstrate to them—especially in the health system—that this is effective.”*

Participants emphasized that short-term external support, while helpful, would not address the deeper structural issues that limit consistent service delivery.

Discussions of sustainability also extended beyond program financing to the affordability of long-term NCD care for patients. Several stakeholders argued that improving medicine availability, expanding insurance coverage, and reducing out-of-pocket expenditures would be necessary to sustain gains in hypertension and diabetes management. Government officials proposed reforms such as broader insurance coverage and increased access to affordable medicines. They cautioned that as NCD burden and treatment complexity grow, addressing spending patterns and patient affordability would become an even more pressing challenge over the next five to seven years.

Beyond financing, stakeholders emphasized the importance of government ownership at the municipal and ward levels. Participants suggested that local leaders, health coordinators, and elected officials would need to recognize the value of community-based NCD services and incorporate them into routine planning and budgets. Across stakeholder groups, sustainability was framed not simply as maintaining supplies and training, but as ensuring that community-based NCD interventions became embedded within existing health system structures and priorities.

Thus, overall, while community-based NCD care was viewed as both needed and potentially acceptable, its implementation was constrained by health workforce shortages, limited resources, and concerns about long-term sustainability. Given these challenges, participants considered whether digital health strategies could complement existing services by supporting follow-up, communication, and patient engagement without substantially increasing demands on an already stretched health system. The final section examines perceptions of mHealth strategies—including SMS and audio messages—and their feasibility in supporting community-based NCD management.

### 6. Feasibility of mHealth strategies

#### Acceptability.

Across stakeholder groups, mHealth strategies were generally viewed as acceptable and potentially useful for supporting medication adherence, appointment reminders, and health education. Community members, FCHVs, FB-CHWs, and organizational stakeholders frequently described mobile communication as a practical way to reinforce health messages and maintain contact with individuals between in-person visits. Participants particularly valued the potential reach of mobile phones, noting that reminders could help overcome challenges associated with missed household visits and competing daily responsibilities. One noted, *“If you send volunteers door-to-door, some houses might not have anyone at home, or they might miss someone. But a mobile phone is almost always present with at least one person in the household. When a message arrives, they look at it once, press it, and even those who can’t read will put it to their ear and listen. They will understand the message and receive the information.”*

However, support for mHealth was largely conditional. Participants consistently emphasized that digital communication should complement rather than replace face-to-face interactions with FCHVs and healthcare workers. One FCHV shared, *“When I send a message to a pregnant woman, it says something like, ‘It’s been this many months, you need to go for a checkup.’ But when I visit her in person, she actually goes.”*

Some participants felt that direct, in-person communication from FCHVs or healthcare providers would be more effective than text messages. They emphasized that face-to-face interactions allow for clarification, personalized advice, and greater accountability. An international organization representative suggested that messages personalized with FCHV names and sent from trusted health institutions could enhance credibility. Several stakeholders described hybrid models combining in-person outreach with digital reminders as more acceptable and potentially effective than either approach alone.

#### Digital access and literacy.

Although participants generally supported mHealth, many questioned whether SMS-based approaches would adequately reach those most affected by NCDs. FCHVs, FB-CHWs, and community members identified limited literacy, shared phone ownership, poverty, and older age as important barriers to engagement with text-based messaging. Participants frequently noted that older adults, the primary target population for hypertension and diabetes management, may have difficulty reading or responding to text messages. “In villages, it’s mostly elderly people who stay behind. Their sons and daughters, who could understand such things, don’t live with them. So, I don’t think text messages will help them,” an FB-CHW noted. Some also highlighted that mobile phone access within households was uneven, creating uncertainty about whether health messages would reach the intended recipient.

Audio or voice-based formats were proposed as a way to overcome literacy barriers and improve accessibility among older adults and individuals with limited formal education. “Audio has been more effective than reading. Even those who cannot read can listen to it on a mobile device and get the information they need,” a community member noted in an FGD, and others agreed. Participants felt that mHealth would be useful only when there was a combination of phone ownership, digital literacy and the ability of users to meaningfully engage with the information provided.

#### Interpersonal communication.

Despite enthusiasm for digital tools, participants consistently viewed interpersonal communication as more influential than technology-based reminders alone. Community members, FCHVs, and FB-CHWs described face-to-face interactions as particularly important for building trust, clarifying health information, and encouraging behavior change.

Many participants expressed a preference for phone calls over text messages because they were viewed as more personal and easier to understand. Others suggested that recorded voice messages from trusted health professionals could improve credibility and engagement. These findings indicate that participants viewed mHealth primarily as a mechanism for reinforcing existing relationships rather than replacing human interaction.

#### System capacity.

Beyond end-user considerations, participants highlighted several operational challenges that could affect long-term implementation. Several participants raised practical questions about who would be responsible for delivering and managing reminders, with many FB-CHWs expressing concern that existing workloads would make manual follow-up difficult.

Stakeholders from government and telecommunications sectors emphasized that sustainable implementation would require more than simply sending messages. Manual approaches, they pointed out, might work temporarily but are not scalable or sustainable for long-term health programs. Participants identified the need for automated delivery systems, patient databases, user communication preferences, delivery tracking mechanisms, consent procedures, and regulatory approvals to support long-term implementation. Concerns about data privacy, user consent, and system maintenance were also raised. These findings suggest that participants viewed the feasibility of mHealth interventions as dependent on both user-level factors and broader health system capacity.

Importantly, health workers and FCHVs noted that mHealth interventions alone could not address the broader challenges faced in NCD management. Persistent issues, including staffing shortages, inconsistent availability of medicines and equipment, and limited training opportunities, were seen as critical barriers that would need to be addressed alongside digital strategies to ensure the feasibility and sustainability of community-based interventions like SCALE-NCD.

Table 1 provides additional participant quotations that support and contextualize the key findings identified in the analysis.

**Table 1:** Additional illustrative quotations supporting key findings from IDIs and FGDs.

Theme	Sub-theme	Illustrative quotation
**Community perspectives on NCDs**	**Perception of NCD risk factors**	“For me, it’s because I wasn’t doing exercise. I ate unhealthy food, and in the evening, I’d sit around… That’s why my blood pressure started to rise.” *(Community member, FGD)* “If we want to eat a little more organic food, it’s difficult to get it. Even if we’re willing to pay, it’s hard to find. Either it is diet or work tension, it contributes to high blood pressure and sugar issues becoming more prominent.” *(Community member, FGD)* “I think it’s hereditary. My parents had it, and now I do too.” *(Community member, FGD)*
	**Barriers to NCD management**	“If I feel unwell, I go [for a check-up]. Otherwise, check-ups cost a lot, so I avoid frequent visits.” *(Community member, FGD)*
		“One patient said, ‘These medicines are too expensive, I won’t buy them,’ and stopped taking diabetes medication.” *(FB-CHW, IDI)* “There’s a common belief that once you start taking hypertension medicine, you have to take it for life. Those who have never gotten checked often say they’ve heard this and avoid starting treatment altogether.” (FB-CHW, IDI)
	**Treatment adherence**	“For diabetes, I don’t eat sugar at all. I have one meal of rice and another of roti. I avoid cold drinks. I used to drink alcohol, but I stopped after being diagnosed with diabetes.” *(Community member, FGD)*
**Health system trust**	**Government health services**	“Don’t take the free medicine… the government’s free medicine is not effective.” *(Community member, FGD)* “I went to the health post recently for medication. A woman staff member said, ‘You can only take one month’s supply,’ even though the rule is for three months under the health insurance scheme. It’s frustrating.” *(Community member, FGD)* “We often don’t know what’s covered under insurance. Staff keep medicines for their acquaintances.” *(Community member, FGD)* “The thing is, if you go to a government hospital to seek consultation, they can’t even speak properly... Even when you go to the hospital, they can’t talk to the patient properly, explain things well, and provide the correct medication.” *(Community member, FGD)*
	**Preference for private care**	“[Government] hospitals are crowded. I go to a private clinic where an army doctor does a complete check-up.” *(Community member, IDI)* “Even those who can’t afford it go… they might even sell a buffalo to pay.” *(Community member, FGD)*
**FCHVs in NCD management**	**Trust and credibility**	“Volunteers can’t help; they spend their days working in cow sheds, and then they come and say, ‘Let’s measure your blood pressure.’ What’s the use?” *(Community member, FGD)* “If the health volunteers visited homes regularly and performed checkups, that would be very helpful.” *(Community member, FGD)*
		“At first they [community members] mocked us… now they seek us out to check blood pressure.” *(FCHV, FGD)* “Initially, they didn’t trust us. But as we started identifying previously unknown illnesses like high blood pressure and diabetes, they realized the importance of our work. Now, they see the benefit and call us for health checks.” *(FCHV, FGD)*
		“Their [FCHVs] job is to inform the community when a program is coming, to let people know. But when it comes to actually conducting the [health] program, that’s our job. They don’t have that capacity. Our volunteers have a little education,” *(FB-CHW, IDI)*
	**Accessibility and community reach**	“In earlier days, during door-to-door programs, people wouldn’t show up when called. FCHVs know every household personally, so they manage to call them somehow. When we go alone, people don’t recognize us,” *(FB-CHW, IDI)*
	**Prerequisites for expanding FCHVs role**	“Sometimes we continue using old methods while many new updates occur. If we receive proper training on hypertension and diabetes, it would enhance our work.” *(FCHV, IDI)* “Maybe a jacket with a logo could be introduced. That way, when we’re working, people in the community can identify us… our bags should be large enough to the materials. Functional and easy to carry.” *(FCHV, FGD)*
		“Providing FCHVs with refresher training would help. Since they are always with the community, they can stay engaged with the people.” *(FB-CHW, IDI)* “It would be beneficial if we, including the FCHVs, received training from time to time. Also, if the glucometers and their kits could be made available continuously, it would help us provide uninterrupted service. If we don’t have the necessary equipment when people come, it sends the message that the service isn’t reliable, and they may not return. So, once a service starts, it should continue consistently.” *(FB-CHW, FGD)*
		“The first and foremost thing would be to provide them [FCHVs] with health education. Once they are educated about these conditions and understand how to become healthy and when to refer someone to a health facility, they can effectively raise awareness in their communities.” *(Stakeholder, IDI)*
	**Sustainability of volunteer model**	“Looking at sustainability, FCHVs might not last long because most of them are not highly educated. In the future, we might not get anyone ready to be FCHVs, as people may not want to volunteer anymore.” *(Stakeholder, IDI)* “Older volunteers have retired... and the center is not recruiting new ones.” *(Stakeholder, IDI)*
**Health system constraints**	**Compensation**	“Public health office gives Rs. 400 per session, right? For some, that might work, but others might need more. What about someone who hires labor for Rs. 800 to work on their maize field while they attend [training]? They’re even spending money on transportation—petrol or vehicle costs.” *(FCHV, FGD)* “While we’re doing this work, it’s hard to manage with just 400. Yesterday, I had to pay a thousand rupees to cut a tree. How can we manage everything with just 400?” *(FCHV, IDI)*
		“The FCHVs haven’t said that the workload is too much. However, they do express a desire to be compensated for the days they work after completing their tasks.” *(FB-CHW, IDI)* “They only get Rs. 400 if they are asked for work. Even the farmers get seven hundred to thousand. FCHVs work on four hundred only. With that amount, it’s obvious that it is very difficult.” *(FB-CHW, IDI)*
	**Training and resource limitations**	“While working, sometimes materials break or wear out. And some things just stop working after a while.” *(FCHV, IDI)*
		“Since we don’t have the means to purchase the kits ourselves, we rely on the government or other organizations to supply them. If they provide the kits, we can offer the service. For instance, there was a time when we experienced a stock-out. Even if we have the machine, we can’t work without the kits.” *(FB-CHW, IDI)* “When we requested more [glucose testing kits] from the metropolitan, they didn’t supply them. What was available before is no longer available now” *(FB-CHW, IDI)*
	**Sustainability and government ownership**	“If we can try to link up with some other projects from other countries or provincial governments, they can also supply additional drugs like metformin, Amlod, and other essential medicines.” *(FB-CHW, IDI)*
		“If we can supply composition-based medicines, or at the metropolitan level establish affordable pharmacies – maybe two or three outlets that offer medicines 20–30% cheaper than other places – or at the national level, expand the scope of insurance then we can make some progress.” *(Stakeholder, IDI)*
		“At the municipal level, not just the staff but also the mayors or municipal chiefs, or health coordinators from the municipalities, should recognize its [FCHV-led NCD intervention] effectiveness… They should think, ‘For this, we need to allocate a budget and communicate with the female health volunteers.’” *(Stakeholder, IDI)*
		“If the program is still running two to three years after you leave, it shows that the program you initiated was effective… Efforts and programs should be implemented to make that accountable government take even more ownership.” *(Stakeholder, IDI)*
**Feasibility of mHealth strategies**	**Acceptability**	“If I get a call saying, ‘It’s time to check your blood pressure,’ that would be helpful. Then you can plan and make time.” *(Community member, FGD)*
		“If a diabetic or hypertensive patient were to miss their medication or check-up, an SMS reminder system could be very effective. People often forget the dates for check-ups or medication pickups. If a message reminds them, they might take better precautions.” *(FCHV, FGD)*
		“Everyone in the household would have the chance to know [health information], including those who can’t visit the health post. Nowadays, almost everyone has a mobile phone.” *(FB-CHW, IDI)*
	**Digital access and literacy**	“Some people don’t know how to read. Some [community members] are very poor. They can’t afford repairing their phones once they get damaged.” *(FB-CHW, IDI)*
	**Interpersonal communication**	“For those of us who might not understand messages, having someone come in person is always better.” *(Community member, FGD)*
		“If we only send a message, the recipients may see it and say, “Oh, it’s just a message,”… However, when the volunteer personally tells them, they feel the need to go [for a check-up]. So, the chances of them responding are much higher.” *(FB-CHW, IDI)* “When we refer a complicated case from our hospital and then follow up with a call to ask about their condition, the patients respond very positively and are happy to hear from us. They feel like we cared enough to call them, which creates a very positive impression.” *(FB-CHW, FGD)*
	**System capacity**	“Manually sending messages might work for the short term. If you want to go for the long term, you need to develop a system... store information about all the people in Pokhara, their mobile numbers, and details about their conditions. Keep track of what information needs to be sent to them, and through which medium it would be most appropriate.” *(Stakeholder, IDI)*

## Discussion

Community-based management of hypertension, diabetes, and smoking is increasingly recognized as s strategy to expand access to care in low resource settings. In this study, we explored how task-sharing through FCHVs and complementary mHealth strategies could support NCD prevention and management, and examined factors shaping feasibility, acceptability, and sustainability. In our study, while community members demonstrated awareness of hypertension, diabetes, and smoking as key health concerns, multiple factors shaped their risk exposure and health-seeking behaviors. Once diagnosed, most participants reported consistent adherence to prescribed medications; however, challenges in sustaining lifestyle modifications and treatment adherence included financial constraints, concerns about long-term medication use, and limited access to services.

Trust in government health services emerged, not surprisingly, as a central determinant of where and how people seek NCD care [21,22]. Stock-outs of essential medicines, perceived poor quality of free medications, and negative patient-provider interactions in government health facilities contribute to a strong preference for private healthcare despite its financial burden. Institutional trust—built through reliable, respectful, and competent system performance—is essential to increasing health care service utilization. In line with WHO guidance emphasizing the importance of leadership and coordinated governance, our findings suggest that strengthening government health services in parallel with community-based efforts can prevent fragmentation and support long-term engagement in care. Task-sharing programs should prioritize clear lines of accountability, active municipal oversight, and mechanisms for local adaptation to ensure community needs are met and to sustain confidence in public health services.

Against this backdrop, FCHVs were recognized as trusted, accessible figures within their communities particularly for maternal and child health, and increasingly, for their role in NCD screening. Initial skepticism towards FCHVs’ ability to conduct blood pressure and glucose monitoring appeared to diminish over time, especially when their screening results were confirmed by FB-CHWs. Importantly, community acceptance of FCHVs in NCD management was closely tied to perceptions of their training, skill level, and ability to explain results and provide appropriate follow-up guidance. Reliable provision of medical equipment and supplies, consistent availability of medications, and robust training programs were seen as critical enablers for both FCHVs and FB-CHWs. This echoes other studies emphasizing the need for training and support for FCHVs [14,22,23]. WHO guidance on implementing task-sharing programs describes effective redistribution of clinical tasks as a capacity building process including training, supervision, and role clarity to maintain service quality and legitimacy. Ensuring that FCHVs are equipped not only with tools and a stable supply chain, but also with communication skills and technical capacity will be essential to the success of task shifting for NCDs in similar peri-urban contexts. Programs should consider structured refresher training, mentorship by facility-based staff, and regular competency assessments to reinforce confidence and credibility among both FCHVs and community members.

While participants in our study strongly supported the expanding role of FCHVs in NCD prevention and screening, concerns around workload, motivation, and sustainability of a voluntary model were raised. Existing research on the FCHV program has highlighted the growing misalignment between expectations of FCHV performance and the limited incentives provided to support their work [24,25]. Recent literature suggests that expectations for volunteers to deliver increasingly time-bound and medically technical services — such as blood pressure screening — without corresponding compensation can erode both morale and legitimacy. While many FCHVs remain motivated by community service, religious merit, and social recognition, stakeholders have noted that expanding responsibilities — including disaster response and NCD screening — may require rethinking incentive structures. Importantly, incentives should be sustainable and transparent, and aligned with both FCHVs’ motivations and evolving health system demands.

The ideal solution is to offer a salary, but even in the absence of a salary, supports such as advanced training, consistent and supportive supervision, reliable availability of supplies, activity-based allowances, retirement stipends, and priority access to government services can be important supports and motivators for CHWs [26–30]. Visible incentives such as transport reimbursement, free healthcare, representation in local health management committees, and public acknowledgment have been noted as ways to support motivation while preserving the community-based ethos of the program [24,31]. Efforts to sustain the FCHV program must consider not only equitable and consistent incentive structures, but also improved supervision, workload boundaries, and respect from the broader health system [32]. As task-sharing for NCDs continues to expand, aligning program demands with FCHVs’ motivations, expectations, and lived experiences will be critical to long-term effectiveness.

In addition to FCHVs providing NCD care, the use of mHealth strategies such as SMS and phone call reminders emerged as a promising but complex intervention component. Digital communication was widely seen as helpful for reminders and behavior change support, but concerns around literacy, mobile phone penetration, and message comprehension raised questions of digital equity. In practice, this implies that SMS-based approaches may be more feasible among younger and more digitally literate populations, while elderly, low-income, and peri-urban populations may require alternative or complementary modalities such as audio-based formats, simplified messages, and multimodal delivery mechanisms to achieve comparable reach and engagement. These concerns align with prior evidence from mHealth interventions in LMICs, which emphasize the importance of user-centered design, technology literacy, and inclusive access strategies for scaling digital health solutions [33,34]. Without such adaptations, digital health solutions risk limited uptake or benefit among the populations most in need of support.

Furthermore, the feasibility and sustainability of mHealth strategies hinges not only on end-user uptake but also on backend infrastructure and institutional coordination. Our findings suggest that message delivery systems require significant setup, automation, and regulatory compliance, including user consent, approval from telecom authorities, and integration with public health tracking systems. These system-level requirements suggest that mHealth interventions may be unlikely to be rapidly deployable or easily scalable in similar settings in the absence of centralized governance and dedicated technical capacity within the public health system. Although cost data were not collected, stakeholders’ concerns imply that the ongoing technical maintenance and cross-sector coordination required may pose challenges to long-term sustainability, particularly if platforms are implemented as parallel or donor-dependent systems. Successful integration of mHealth into community-based NCD care will depend on addressing both technological and human-level barriers — ensuring that digital strategies complement the interpersonal trust and outreach provided by FCHVs.

Finally, a key theme that emerged in this study is that long-term sustainability hinges on systemic integration and government ownership. While donor-funded programs can catalyze innovation, they often risk collapse unless institutionalized within local systems. To ensure continuity, stakeholders such as municipal mayors, ward chairs, and health coordinators should be engaged early—not just as implementers, but as long-term stewards [25]. Taken together, our findings underscore that governance and leadership, financing, workforce support, and service delivery are interdependent factors that determine the feasibility and sustainability of task-sharing interventions. Programs should ensure integrated planning across these elements, with active monitoring and adaptation to local contexts.

This study has several limitations. Given the sensitive nature of discussing motivation, performance, and trust in government health systems, responses may have been influenced by social desirability bias, particularly among FCHVs, facility-based health workers, and government-affiliated stakeholders. Although we sought to mitigate this through assurances of confidentiality, neutral and open-ended questioning, and triangulation across participant groups and data collection methods, some degree of positive reporting or underreporting of critical perspectives cannot be ruled out. Nonetheless, the convergence of findings across FGDs and IDIs and across diverse stakeholder groups strengthens confidence in the credibility of the results.

These findings have several implications for task-sharing and mHealth as strategies for NCD care in peri-urban and metropolitan areas of Nepal, and may be informative for similar settings elsewhere. Both task-sharing with FCHVs and mHealth strategies can expand reach and support adherence, but special attention is needed to reach populations at risk of being left behind — such as lower-income individuals and those in peri-urban or remote areas — who face financial, geographic and digital barriers to care. Strengthening government health services, improving medicine availability, and investing in patient-centered care are critical complements of community-based efforts and help rebuild trust in public health systems. Addressing workforce shortages, ensuring equipment availability, and developing supportive supervision structures will be essential to sustain task-sharing models. And importantly, building a supportive environment for FCHVs—through tools, training, recognition, and community legitimacy—remains foundational to intervention success. Similarly, successful mHealth implementation depends on user-centered design, accessible formats, backend infrastructure, and integration with existing health systems to avoid parallel or donor-dependent platforms. Task-sharing and mHealth interventions should therefore not be viewed as standalone solutions, but as components of a more comprehensive system-strengthening effort, with careful attention to digital equity and implementation infrastructure.

As Nepal confronts a growing burden of NCDs, integrating community health workers like FCHVs and mHealth strategies into NCD prevention and management may represent a potentially valuable strategy to expand reach and improve health equity in peri-urban metropolitan settings, under appropriate system conditions. Investments in public sector capacity, clear role delineation, supportive supervision, and sustainable incentive structures will be critical to ensuring that community-based NCD interventions are both effective and equitable over the long term.

## Supporting information

S1 Questionnaire Inclusivity in global research. (DOCX)
