# Supplementary material for: Non-communicable disease care in peri-urban Nepal: Potential for community-based interventions
Source: PLOS Glob Public Health. 2026 Jul 15;6(7):e0005015. doi: 10.1371/journal.pgph.0005015 (PMC13372126; doi:10.1371/journal.pgph.0005015)
Supplement: Attachment — Submitted filename: Response to Reviewers.docx [file pgph.0005015.s003.docx]

| **Reviewer 1** | |
| --- | --- |
| **Abstract**  1. First need to mention that NCD is a growing problem in Nepal and that there is increasing risk associated with several risk factors. | Thank you for this suggestion. We have revised the opening sentence of the abstract to explicitly highlight the growing burden of NCDs in Nepal and the increasing exposure to key risk factors, including hypertension, diabetes, and tobacco use. |
| 2. Briefly state who FCHVs are and what they are currently doing. | We have revised the abstract to briefly define FCHVs as a national cadre of volunteer community health workers primarily engaged in maternal and child health promotion, providing context for their proposed role in community-based NCD care. |
| 3. Line 30- What do you mean by organizational representatives? | Thank you for this comment. We have clarified this term in the abstract to specify that organizational representatives included participants from government, public health, academic, international, and telecommunications sectors. These individuals represented institutions involved in NCD policy, service delivery, research, advocacy, and digital health implementation. |
| 4. Tighten the recommendations, now it looks very broad | We have revised the concluding sentence of the abstract to make the recommendations more specific and closely aligned with the study findings. The revised text emphasizes concrete implementation levers (training and supervision, supply availability and incentives, and trust-building through consistent service delivery). |
| **Introduction**  1. You should use a reference for the first sentence | Added, thank you. |
| 2. Detail out a bit more around community health workers globally and how they can contribute to management of chronic disease using a community-based approach. | We have included additional context on the role of community health workers globally, highlighting their contribution to chronic disease prevention and management through community-based approaches such as screening, counseling, adherence support, and referral. This added text situates the Nepal FCHV model within the broader global evidence base on task-sharing and community-based NCD care. |
| 3. More perspective is required, such as around government health system trust issues associated with FHCV intervention in NCD management in Nepal. | We thank the reviewer for this suggestion. We have added discussion of trust in government health systems as a critical factor influencing FCHV-led NCD care. |
| 4. Lines 68-74: Why you bring forward mHealth issues here? Is the FCHV delivers interventions using mHealth? Need clarity around this issues. | We have clarified that mHealth is a complementary strategy to support task-sharing with FCHVs in the SCALE-NCD intervention. mHealth approaches, such as SMS reminders explored in our study, may enhance patient follow-up and reinforce health information provided during FCHV home visits. |
| 5. I do not see in objective statement for the research rather findings are presented in the last paragraph of Introduction. Please state your objective and rationality for the research in the last paragraph. | Thank you, we have added a clear objective statement at the end of the Introduction. The study aims to explore structural, social, and contextual factors influencing FCHV-led NCD care and the feasibility of complementary mHealth strategies, with the rationale of informing scalable, contextually appropriate task-sharing models. |
| **Methods**  1. How are the participants recruited? What are the eligibility criteria for selection? Need more details on participant recruitment. | We have added detailed information on participant recruitment and eligibility. Participants were recruited using a purposive and convenience sampling strategy to ensure representation of diverse perspectives across community members, FCHVs, facility-based CHWs, and key organizational representatives. Inclusion criteria for each group have been specified to clarify selection. |
| 2. Methods section is inadequate. More detailed description of data collection, management and analysis is required. | We have expanded the Methods section to provide a comprehensive description. We have added key details on data collection, management, and analysis. This includes use of semi-structured interview guides tailored to each participant group, transcription and translation of interviews, integration of field notes, and thematic analysis using an inductive and deductive approach with iterative codebook development and inter-coder reliability checks. |
| **Results**  Results seems to be quite elaborative but scattered. It would be good to structure the result using any framework such as Socio-ecological model. | We thank the reviewer for this suggestion. Our results were carefully structured through an iterative process based on the thematic analysis and fieldwork insights, to avoid omitting important domains. Following Reviewer 2’s suggestion, we focused on aligning the discussion with the WHO guidance on task-sharing implementation to strengthen interpretative depth, while retaining the results’ structure as it reflects the voices and experiences of participants. |
| **Discussion**  The discussion section looks mostly focusing on the implementation and sustainability of FCHVs in NCD management which is good. But discussion needs to be aligned with the result section. I would ask the authors to put more thoughts around structuring discussion and results which are aligned together. | We appreciate the reviewer’s comment. The discussion is intentionally aligned with the results, as the paper’s primary focus is on the implementation and sustainability of FCHVs in NCD management. Each element discussed, including barriers to management, health system trust, FCHVs role, system constraints (compensation/incentives, training and resources, sustainability) and mHealth feasibility, directly correspond to the results sections. We have reviewed the discussion carefully to ensure that all major findings from the results are interpreted and contextualized. |
| **Reviewer 2** |  |
| 1. The manuscript highlights several systemic and structural challenges, particularly around sustainability of the FCHV volunteer model, supply chain gaps, and mistrust in public services. However, the final conclusions frame community-based task-sharing as highly promising without sufficiently addressing these constraints. Reframe conclusions to more explicitly acknowledge these limitations and avoid overgeneralization of feasibility. | Thank you for your comment. We have revised the concluding section of the Discussion to more explicitly acknowledge the structural and systemic constraints identified in the study, including reliance on a voluntary FCHV workforce, supply chain limitations, and mistrust in public health services. The conclusions now emphasize that the feasibility of community-based task-sharing and mHealth interventions is conditional on broader health system strengthening. |
| 2. While thematic analysis is well-described, the interpretation remains largely descriptive. A guiding framework (e.g., WHO Task-Sharing Implementation Framework, Health System Trust Framework, or CHW Motivation Theory) could deepen the explanatory power of the findings. Introduce a framework in the methodology and use it to structure the discussion. | Thank you for this helpful suggestion. We retained the structure of the discussion based on how the results naturally flowed, as this allowed us to include all key domains identified in our thematic analysis. We integrated components of the WHO guidance on task-sharing implementation where relevant—governance and leadership, access to commodities, service delivery, health workforce training, and financing—to provide interpretive depth and link our findings to established evidence. |
| 3. The manuscript does not clearly describe recruitment procedures, sampling rationale, or how thematic saturation was determined. These are essential for qualitative rigor. Add details on inclusion criteria, participant selection methods, and saturation assessment. | Thank you, we have clarified recruitment procedures and sampling rationale, including purposive selection to reflect diverse perspectives. Thematic saturation is now described as the point at which no new themes emerged across interviews or focus groups. Inclusion criteria for all participant groups has also been added. |
| 4. Given the sensitive nature of discussing motivation, performance, or institutional trust, social desirability bias is likely. Acknowledge and discuss this limitation explicitly in the Methods or Discussion. | Thank you for this point. We have added a discussion of potential social desirability bias in the Discussion section. |
| 5. The study was conducted in a single metropolitan area, but some statements are generalized to the national level. Clarify contextual boundaries and avoid sweeping claims beyond the study setting. | Thank you for this important point. We have revised the Discussion to more clearly anchor interpretations to the peri-urban metropolitan study setting. Where relevant, we have reframed broader statements as implications for comparable settings rather than national-level conclusions. These revisions clarify the contextual boundaries of the findings while preserving their relevance for settings with similar health system and implementation characteristics. |
| 6. The section on mHealth perceptions is informative, but the implications remain unclear. Discuss feasibility in terms of digital literacy, system infrastructure, and cost sustainability. | We have expanded the Discussion to more explicitly address the feasibility implications of mHealth strategies. This synthesis clarifies that mHealth approaches are most feasible as complementary, hybrid strategies rather than standalone interventions. |
| 7. Consider adding a table summarizing key themes and representative quotations to improve readability. | We have added a table at the end of the Results section presenting representative quotations aligned with each major theme and sub-theme. |
