# Supplementary material for: Non-communicable disease care in peri-urban Nepal: Potential for community-based interventions
Source: PLOS Glob Public Health. 2026 Jul 15;6(7):e0005015. doi: 10.1371/journal.pgph.0005015 (PMC13372126; doi:10.1371/journal.pgph.0005015)
Supplement: Attachment — Submitted filename: Response_to_Reviewers_auresp_2.docx [file pgph.0005015.s004.docx]

**Response:**

We thank the editor for this helpful guidance. We have substantially revised and reorganized the presentation of findings within the results sections to better highlight what was learned relative to the study objectives and to strengthen the analytic presentation of the data.

Specifically, we have:

- Significantly condensed the in-text presentation of findings and removed descriptive material that did not directly address the study objectives.
- Reorganized several subsections (specifically Sections 4-6) to present a clearer analytic narrative regarding the factors influencing the delivery, feasibility, and sustainability of community-based NCD interventions involving FCHVs.
  - Revised the FCHV section findings to emphasize key themes, including trust and credibility, accessibility and community reach, prerequisites for role expansion, and sustainability of the volunteer model.
  - Reorganized the subsequent health system constraints section to focus on the structural conditions required for successful implementation and scale-up of FCHV-led NCD care, including compensation, training and resource limitations, sustainability, and government ownership.
  - Revised the mHealth section to focus on feasibility and implementation considerations, including acceptability, digital access and literacy, interpersonal communication preferences, and health system capacity requirements.
- Revised Table 1 to align with the updated structure and provide additional supporting quotations for each major finding. Moved majority of the illustrative quotations from the main text to Table 1 and retained only a limited number of representative quotations within the results section.

The progression of sections builds on one another: we first present evidence of a growing NCD burden and awareness alongside persistent gaps in screening, treatment uptake, and long-term management, as well as widespread concerns regarding health system accessibility and trust. We then examine the potential role of FCHVs in addressing these gaps, followed by the health system requirements necessary for successful implementation and sustainability of FCHV-led community-based NCD management, including training, resources, compensation, and government ownership. Finally, we assess the feasibility and acceptability of mHealth strategies as complementary approaches to support community-based NCD management.

These revisions were intended to present a more focused analysis of the structural, social, and contextual factors influencing the implementation of community-based NCD interventions with complementary mHealth strategies like SCALE-NCD, consistent with the objectives of the study. We believe the revised results section now addresses the reviewers’ suggestions.
