# Supplementary material for: Non-communicable disease care in peri-urban Nepal: Potential for community-based interventions
Source: PLOS Glob Public Health. 2026 Jul 15;6(7):e0005015. doi: 10.1371/journal.pgph.0005015 (PMC13372126; doi:10.1371/journal.pgph.0005015)
Supplement: Peer review history

# Peer review: Decision Letter 0

**DOI:** 10.1371/journal.pgph.0005015.r001

**Authors:** Katherine Kokkinias

12 Nov 2025

PGPH-D-25-01991

Non-communicable disease care in peri-urban Nepal: Potential for community-based interventions

PLOS Global Public Health

Dear Dr. Neupane,

Thank you for submitting your manuscript to PLOS Global Public Health. After careful consideration, we feel that it has merit but does not fully meet PLOS Global Public Health’s publication criteria as it currently stands. Therefore, we invite you to submit a revised version of the manuscript that addresses the points raised during the review process.

==============================

The manuscript has been evaluated by two reviewers, and their comments are available below.

The reviewers have raised a number of major concerns. The reviewers would like more details regarding the methodology of the study. Specifically, please describe the recruitment process, sampling rational, how thematic saturation was determined, and data analysis. The reviewers provided suggestions to strength the introduction and discussion. They also suggest tempering conclusions and mentioning limitations such as desirability bias.

Could you please carefully revise the manuscript to address all comments raised?

==============================

Please submit your revised manuscript by Dec 25 2025 11:59PM. If you will need more time than this to complete your revisions, please reply to this message or contact the journal office at globalpubhealth@plos.org. When you're ready to submit your revision, log on to https://www.editorialmanager.com/pgph/ and select the 'Submissions Needing Revision' folder to locate your manuscript file.

Please include the following items when submitting your revised manuscript:

- A rebuttal letter that responds to each point raised by the editor and reviewer(s). You should upload this letter as a separate file labeled 'Response to Reviewers'.
- A marked-up copy of your manuscript that highlights changes made to the original version. You should upload this as a separate file labeled 'Revised Manuscript with Track Changes'.
- An unmarked version of your revised paper without tracked changes. You should upload this as a separate file labeled 'Manuscript'.

Guidelines for resubmitting your figure files are available below the reviewer comments at the end of this letter.

We look forward to receiving your revised manuscript.

Kind regards,

Katherine Demi Kokkinias, Ph.D.

Staff Editor

PLOS Global Public Health

Journal Requirements:

1. Please include a complete copy of PLOS’ questionnaire on inclusivity in global research in your revised manuscript. Our policy for research in this area aims to improve transparency in the reporting of research performed outside of researchers’ own country or community. The policy applies to researchers who have travelled to a different country to conduct research, research with Indigenous populations or their lands, and research on cultural artefacts. The questionnaire can also be requested at the journal’s discretion for any other submissions, even if these conditions are not met. Please find more information on the policy and a link to download a blank copy of the questionnaire here: https://journals.plos.org/globalpublichealth/s/best-practices-in-research-reporting. Please upload a completed version of your questionnaire as Supporting Information when you resubmit your manuscript.

2. In the online submission form, you indicated that Full interview and focus group transcripts cannot be shared publicly to protect participant confidentiality. Relevant de-identified excerpts are included within the paper. Additional anonymized data supporting the findings of this study are available from the corresponding author (DN) upon reasonable request.

All PLOS journals now require all data underlying the findings described in their manuscript to be freely available to other researchers, either

1. In a public repository,

2. Within the manuscript itself, or

3. Uploaded as supplementary information.

This policy applies to all data except where public deposition would breach compliance with the protocol approved by your research ethics board. If your data cannot be made publicly available for ethical or legal reasons (e.g., public availability would compromise patient privacy), please explain your reasons by return email and your exemption request will be escalated to the editor for approval. Your exemption request will be handled independently and will not hold up the peer review process, but will need to be resolved should your manuscript be accepted for publication. One of the Editorial team will then be in touch if there are any issues.

3. If the reviewer comments include a recommendation to cite specific previously published works, please review and evaluate these publications to determine whether they are relevant and should be cited. There is no requirement to cite these works unless the editor has indicated otherwise.

[Note: HTML markup is below. Please do not edit.]

Reviewers' comments:

Reviewer's Responses to Questions

**Comments to the Author**

1. Does this manuscript meet PLOS Global Public Health’s publication criteria? Is the manuscript technically sound, and do the data support the conclusions? The manuscript must describe methodologically and ethically rigorous research with conclusions that are appropriately drawn based on the data presented.

Reviewer #1: Partly

Reviewer #2: Partly

**********

2. Has the statistical analysis been performed appropriately and rigorously?

Reviewer #1: N/A

Reviewer #2: N/A

**********

3. Have the authors made all data underlying the findings in their manuscript fully available (please refer to the Data Availability Statement at the start of the manuscript PDF file)?

The PLOS Data policy requires authors to make all data underlying the findings described in their manuscript fully available without restriction, with rare exception. The data should be provided as part of the manuscript or its supporting information, or deposited to a public repository. For example, in addition to summary statistics, the data points behind means, medians and variance measures should be available. If there are restrictions on publicly sharing data—e.g. participant privacy or use of data from a third party—those must be specified.

Reviewer #1: Yes

Reviewer #2: Yes

**********

4. Is the manuscript presented in an intelligible fashion and written in standard English?

PLOS Global Public Health does not copyedit accepted manuscripts, so the language in submitted articles must be clear, correct, and unambiguous. Any typographical or grammatical errors should be corrected at revision, so please note any specific errors here.

Reviewer #1: Yes

Reviewer #2: Yes

**********

5. Review Comments to the Author

Please use the space provided to explain your answers to the questions above. You may also include additional comments for the author, including concerns about dual publication, research ethics, or publication ethics. (Please upload your review as an attachment if it exceeds 20,000 characters)

Reviewer #1: Thanks for the opportunity to review the manuscript. While it has sufficient merit and it addresses an important issue it requires major revision to be scientifically suitable for publication. My specific comments for consideration is below:

Abstract

1. First need to mention that NCD is a growing problem in Nepal and that there is increasing risk associated with several risk factors.

2. Briefly state who FCHVs are and what they are currently doing.

3. Line 30- What do you mean by organizational representatives?

4. Tighten the recommendations, now it looks very broad

Introduction

1. You should use a reference for the first sentence

2. Detail out a bit more around community health workers globally and how they can contribute to management of chronic disease using a community-based approach.

3. More perspective is required, such as around government health system trust issues associated with FHCV intervention in NCD management in Nepal.

4. Lines 68-74: Why you bring forward mHealth issues here? Is the FCHV delivers interventions using mHealth? Need clarity around this issues.

5. I do not see in objective statement for the research rather findings are presented in the last paragraph of Introduction. Please state your objective and rationality for the research in the last paragraph.

Methods

1. How are the participants recruited? What are the eligibility criteria for selection? Need more details on participant recruitment.

2. Methods section is inadequate. More detailed description of data collection, management and analysis is required.

Results

Results seems to be quite elaborative but scattered. It would be good to structure the result using any framework such as Socio-ecological model.

Discussion

The discussion section looks mostly focusing on the implementation and sustainability of FCHVs in NCD management which is good. But discussion needs to be aligned with the result section. I would ask the authors to put more thoughts around structuring discussion and results which are aligned together.

Reviewer #2: Dear authors,

Hello

Thank you for the opportunity to review this valuable and timely manuscript. The topic of community-based approaches to non-communicable disease care in peri-urban Nepal is highly relevant, and the study provides rich qualitative insights from multiple stakeholders. The manuscript has several notable strengths, including the relevance of the research question, inclusion of diverse participant groups, and use of authentic, contextualized qualitative data. However, there are a number of areas where clarification and revision would strengthen the manuscript. My major and minor comments are outlined below.

Major Comments

1. The manuscript highlights several systemic and structural challenges, particularly around sustainability of the FCHV volunteer model, supply chain gaps, and mistrust in public services. However, the final conclusions frame community-based task-sharing as highly promising without sufficiently addressing these constraints. Reframe conclusions to more explicitly acknowledge these limitations and avoid overgeneralization of feasibility.

2. While thematic analysis is well-described, the interpretation remains largely descriptive. A guiding framework (e.g., WHO Task-Sharing Implementation Framework, Health System Trust Framework, or CHW Motivation Theory) could deepen the explanatory power of the findings. Introduce a framework in the methodology and use it to structure the discussion.

3. The manuscript does not clearly describe recruitment procedures, sampling rationale, or how thematic saturation was determined. These are essential for qualitative rigor. Add details on inclusion criteria, participant selection methods, and saturation assessment.

4. Given the sensitive nature of discussing motivation, performance, or institutional trust, social desirability bias is likely. Acknowledge and discuss this limitation explicitly in the Methods or Discussion.

5. The study was conducted in a single metropolitan area, but some statements are generalized to the national level. Clarify contextual boundaries and avoid sweeping claims beyond the study setting.

6. The section on mHealth perceptions is informative, but the implications remain unclear. Discuss feasibility in terms of digital literacy, system infrastructure, and cost sustainability.

7. Consider adding a table summarizing key themes and representative quotations to improve readability.

This is a meaningful and well-conceived study that makes an important contribution to understanding the opportunities and challenges for community-based NCD care in Nepal. Addressing the points above, particularly around alignment of conclusions with data, methodological transparency, and incorporation of a theoretical framework, will considerably strengthen the manuscript.

I appreciate your efforts and look forward to seeing the revised version.

Sincerely,

Reviewer

**********

6. PLOS authors have the option to publish the peer review history of their article (what does this mean?). If published, this will include your full peer review and any attached files.

**Do you want your identity to be public for this peer review?**  If you choose “no”, your identity will remain anonymous but your review may still be made public.

For information about this choice, including consent withdrawal, please see our Privacy Policy.

Reviewer #1: No

Reviewer #2: No

**********

[NOTE: If reviewer comments were submitted as an attachment file, they will be attached to this email and accessible via the submission site. Please log into your account, locate the manuscript record, and check for the action link "View Attachments". If this link does not appear, there are no attachment files.]

-->Figure Resubmissions:

While revising your submission, we strongly recommend that you use PLOS’s NAAS tool (https://ngplosjournals.pagemajik.ai/artanalysis) to test your figure files. NAAS can convert your figure files to the TIFF file type and meet basic requirements (such as print size, resolution), or provide you with a report on issues that do not meet our requirements and that NAAS cannot fix.

After uploading your figures to PLOS’s NAAS tool - https://ngplosjournals.pagemajik.ai/artanalysis, NAAS will process the files provided and display the results in the "Uploaded Files" section of the page as the processing is complete. If the uploaded figures meet our requirements (or NAAS is able to fix the files to meet our requirements), the figure will be marked as "fixed" above. If NAAS is unable to fix the files, a red "failed" label will appear above. When NAAS has confirmed that the figure files meet our requirements, please download the file via the download option, and include these NAAS processed figure files when submitting your revised manuscript.

# Author comment: Author response to Decision Letter 1

**DOI:** 10.1371/journal.pgph.0005015.r002

19 Feb 2026

Attachment Submitted filename: Response to Reviewers.docx

# Editor report: Decision Letter 1

**DOI:** 10.1371/journal.pgph.0005015.r003

**Authors:** Katherine Kokkinias, Graeme Hoddinott

29 Apr 2026

PGPH-D-25-01991R1

Non-communicable disease care in peri-urban Nepal: Potential for community-based interventions

PLOS Global Public Health

Dear Dr. Neupane,

Thank you for submitting your manuscript to PLOS Global Public Health. After careful consideration, we feel that it has merit but does not fully meet PLOS Global Public Health’s publication criteria as it currently stands. Therefore, we invite you to submit a revised version of the manuscript that addresses the points raised during the review process.

==============================

EDITOR:

In the previous review, both reviewers requested substantial refinement of the findings / results section. While your response to their comments and the sentiment of staying true to what participants reported is noted, the section still reads as overly descriptive and lacking in analysis. It reads like an overall report of the first-level descriptive analysis / thematic organisation of the data, and *not* like a critical *analysis* of what was learnt relative to the objectives that you state. My strong suggestion is that you engage the reviewers' comments on this section more thoroughly. Specifically, move more of the illustrative quotations into Table 1 to support your analytic argument, and then dramatically shorten the in-text representation of the findings to be answering the stated objectives. I believe that this revision is minor, editorial re-organisation of the findings rather than substantive / major. I hope that you are encouraged to complete it.

==============================

Please submit your revised manuscript by May 29 2026 11:59PM. If you will need more time than this to complete your revisions, please reply to this message or contact the journal office at globalpubhealth@plos.org. When you're ready to submit your revision, log on to https://www.editorialmanager.com/pgph/ and select the 'Submissions Needing Revision' folder to locate your manuscript file.

Please include the following items when submitting your revised manuscript:

- A letter that responds to each point raised by the editor and reviewer(s). You should upload this letter as a separate file labeled 'Response to Reviewers'.
- A marked-up copy of your manuscript that highlights changes made to the original version. You should upload this as a separate file labeled 'Revised Manuscript with Track Changes'.
- An unmarked version of your revised paper without tracked changes. You should upload this as a separate file labeled 'Manuscript'.

Guidelines for resubmitting your figure files are available below the reviewer comments at the end of this letter.

As the corresponding author, your ORCID iD is verified in the submission system and will appear in the published article. PLOS supports the use of ORCID, and we encourage all coauthors to register for an ORCID iD and use it as well. Please encourage your coauthors to verify their ORCID iD within the submission system before final acceptance, as unverified ORCID iDs will not appear in the published article. *Only*  the individual author can complete the verification step; PLOS staff *cannot*  verify ORCID iDs on behalf of authors.

We look forward to receiving your revised manuscript.

Kind regards,

Graeme Hoddinott, Ph.D

Academic Editor

PLOS Global Public Health

**Journal Requirements:**

If the reviewer comments include a recommendation to cite specific previously published works, please review and evaluate these publications to determine whether they are relevant and should be cited. There is no requirement to cite these works unless the editor has indicated otherwise.

Please review your reference list to ensure that it is complete and correct. If you have cited papers that have been retracted, please include the rationale for doing so in the manuscript text, or remove these references and replace them with relevant current references. Any changes to the reference list should be mentioned in the rebuttal letter that accompanies your revised manuscript. If you need to cite a retracted article, indicate the article’s retracted status in the References list and also include a citation and full reference for the retraction notice.

**Additional Editor Comments (if provided):**

[Note: HTML markup is below. Please do not edit.]

**Reviewers' comments:**

[NOTE: If reviewer comments were submitted as an attachment file, they will be attached to this email and accessible via the submission site. Please log into your account, locate the manuscript record, and check for the action link "View Attachments". If this link does not appear, there are no attachment files.]

**Figure Resubmissions:**

While revising your submission, we strongly recommend that you use PLOS’s NAAS tool (https://ngplosjournals.pagemajik.ai/artanalysis) to test your figure files. NAAS can convert your figure files to the TIFF file type and meet basic requirements (such as print size, resolution), or provide you with a report on issues that do not meet our requirements and that NAAS cannot fix.

After uploading your figures to PLOS’s NAAS tool - https://ngplosjournals.pagemajik.ai/artanalysis, NAAS will process the files provided and display the results in the "Uploaded Files" section of the page as the processing is complete. If the uploaded figures meet our requirements (or NAAS is able to fix the files to meet our requirements), the figure will be marked as "fixed" above. If NAAS is unable to fix the files, a red "failed" label will appear above. When NAAS has confirmed that the figure files meet our requirements, please download the file via the download option, and include these NAAS processed figure files when submitting your revised manuscript.

# Author comment: Author response to Decision Letter 2

**DOI:** 10.1371/journal.pgph.0005015.r004

5 Jun 2026

Attachment Submitted filename: Response_to_Reviewers_auresp_2.docx

# Editor report: Decision Letter 2

**DOI:** 10.1371/journal.pgph.0005015.r005

**Authors:** Katherine Kokkinias, Graeme Hoddinott, Graeme Hoddinott

25 Jun 2026

Non-communicable disease care in peri-urban Nepal: Potential for community-based interventions

PGPH-D-25-01991R2

Dear Dr. Neupane,

We are pleased to inform you that your manuscript 'Non-communicable disease care in peri-urban Nepal: Potential for community-based interventions' has been provisionally accepted for publication in PLOS Global Public Health.

Before your manuscript can be formally accepted you will need to complete some formatting changes, which you will receive in a follow up email. A member of our team will be in touch with a set of requests.

Please note that your manuscript will not be scheduled for publication until you have made the required changes, so a swift response is appreciated.

IMPORTANT: The editorial review process is now complete. PLOS will only permit corrections to spelling, formatting or significant scientific errors from this point onwards. Requests for major changes, or any which affect the scientific understanding of your work, will cause delays to the publication date of your manuscript.

If your institution or institutions have a press office, please notify them about your upcoming paper to help maximize its impact. If they'll be preparing press materials, please inform our press team as soon as possible -- no later than 48 hours after receiving the formal acceptance. Your manuscript will remain under strict press embargo until 2 pm Eastern Time on the date of publication. For more information, please contact globalpubhealth@plos.org.

Thank you again for supporting Open Access publishing; we are looking forward to publishing your work in PLOS Global Public Health.

Best regards,

Graeme Hoddinott, Ph.D

Academic Editor

PLOS Global Public Health

***********************************************************

Reviewer Comments (if any, and for reference):
